# Fighting pathogenic yeasts with plant defensins and anti-fungal proteins from fungi

**DOI:** 10.1007/s00253-024-13118-1

**Published:** 2024-03-27

**Authors:** Paloma Manzanares, Moisés Giner-Llorca, Jose F. Marcos, Sandra Garrigues

**Affiliations:** Food Biotechnology Department, Instituto de Agroquímica y Tecnología de Alimentos (IATA), Consejo Superior de Investigaciones Científicas (CSIC), Paterna, Valencia Spain

**Keywords:** Plant defensins, Anti-fungal proteins (AFPs), Anti-yeast potency, Mode of action, Biotechnological production

## Abstract

**Abstract:**

Fungal infections represent a significant health risk worldwide. Opportunistic infections caused by yeasts, particularly by *Candida* spp. and their virulent emerging isolates, have become a major threat to humans, with an increase in fatal cases of infections attributed to the lack of effective anti-yeast therapies and the emergence of fungal resistance to the currently applied drugs. In this regard, the need for novel anti-fungal agents with modes of action different from those currently available is undeniable. Anti-microbial peptides (AMPs) are promising candidates for the development of novel anti-fungal biomolecules to be applied in clinic. A class of AMPs that is of particular interest is the small cysteine-rich proteins (CRPs). Among CRPs, plant defensins and anti-fungal proteins (AFPs) of fungal origin constitute two of the largest and most promising groups of CRPs showing anti-fungal properties, including activity against multi-resistant pathogenic yeasts. In this review, we update and compare the sequence, structure, and properties of plant defensins and AFPs with anti-yeast activity, along with their in vitro and in vivo potency. We focus on the current knowledge about their mechanism of action that may lead the way to new anti-fungals, as well as on the developments for their effective biotechnological production.

**Key points:**

*• Plant defensins and fungal AFPs are alternative anti-yeast agents*

*• Their multi-faceted mode of action makes occurrence of resistance rather improbable*

*• Safe and cost-effective biofactories remain crucial for clinical application*

## Introduction

The fungal kingdom includes millions of species, some of which are pathogenic for plants and animals. Fungal infections represent an important risk to human health and food production and safety (Fisher et al. 2020). In medicine, mycoses have hugely increased due to the growing number of immunosuppressive therapies and diseases. In agriculture, fungi are the main pathogens of the crops used for food and feed production. Moreover, mycotoxins produced by some fungi represent a threat for food safety, as they can contaminate food and be dangerous to human health (Liu et al. 2020). Despite this emerging threat, very few classes of anti-fungal agents have been introduced over the last 30 years, with four major classes available to date: azoles, polyenes, echinocandins, and fluorinated pyrimidine (Roemer and Krysan 2014). In addition, there is an alarming appearance of new strains that are resistant to these commonly used anti-fungals, favored by the cross-resistance between drugs used in clinics and fungicides used in agriculture (Perfect 2016). Those resistant fungi include variants of previously susceptible pathogens such as *Aspergillus fumigatus* and multi-resistant emerging species such as *Candida auris* (Fisher et al. 2022), which are currently being considered as critical fungal pathogens in the World Health Organisation (WHO)’s priority pathogens list intended to guide research, development, and public health action (WHO 2022). In this sense, opportunistic infections caused by yeasts, particularly by members of the *Candida* genus but also other emerging species, i.e., *Rhodotorula, Hansenula, Malassezia,* and *Saccharomyces* (Miceli et al. 2011), have become a major threat to humans, with an increase in fatal cases of infections being attributed to the lack of precise anti-yeast therapies and the emergence of resistance (Pote et al. 2020). Therefore, the need for new anti-fungal agents with modes of action different from those currently available is undeniable.

Anti-microbial peptides (AMPs) are a broad class of peptides and small proteins produced by organisms all along the phylogenetic scale (Brogden 2005; Zasloff 2002). AMPs have been proposed as promising candidates for the development of novel anti-microbial compounds (Marcos et al. 2008; Montesinos 2007). A class of AMPs that is of particular interest is the small cysteine-rich proteins (CRPs). These are peptides and small proteins containing multiple cysteine residues that form disulfide bonds and fold into compact structures, conferring a high degree of stability against adverse biochemical and biophysical conditions. Defensins and defensin-like proteins found in mammals, insects, plants, and fungi form by far the largest family of CRPs with anti-microbial activity. This review focuses only on plant and fungal CRPs. Defensins from invertebrate and vertebrate animals have been extensively reviewed elsewhere (Aerts et al. 2008; Hegedüs and Marx 2013; Koehbach 2017; Xu and Lu 2020). Plant defensins compose a numerous group of small cationic CRPs (45–54 amino acids in length) that typically include eight cysteines and four intramolecular disulfide bonds. They are ubiquitous throughout the plant kingdom as part of the innate immunity against microbial infections (van der Weerden and Anderson 2013). Another CRP group of interest comprises the anti-fungal proteins (AFPs) of fungal origin. AFPs are small (45–57 amino acids) and cationic defensin-like proteins that are produced and secreted to the culture medium by filamentous ascomycetes and exhibit anti-fungal activity (Hegedüs and Marx 2013). In general, plant defensins and AFPs show inhibitory activity against both plant and human pathogens, mainly of fungal nature, but occasionally bacterial and in some cases against virus (Garrigues et al. 2018; Hajji et al. 2010; Huber et al. 2018; Sathoff and Samac 2019), and show no toxicity to plants or animal cells (Hegedüs and Marx 2013; Parisi et al. 2019b; van der Weerden and Anderson 2013).

The anti-fungal activity of plant defensins and AFPs also extends to (pathogenic) yeasts. Some of them are potent anti-yeast agents against the model fungus *Saccharomyces cerevisiae* or even against life-threatening *Candida* species. Overall, plant defensins and AFPs show a multi-target mechanism of action different from those of the traditional anti-fungals, making fungal isolates less likely to overcome their inhibitory action and, therefore, limiting the appearance of acquired resistance (Thevissen et al. 2007; van der Weerden et al. 2023).

This review will focus on plant defensins and AFPs active against *Candida* spp. — especially *C. albicans* — and *S. cerevisiae*. Firstly, we will briefly address the sequence and structure of anti-yeast defensins and AFPs, as well as their in vitro and in vivo potency. Links between sequence motifs and activity will be also highlighted where appropriate. Finally, we will discuss the knowledge about their mechanism of action, emphasizing those elucidated in model yeasts, and their biotechnological production, which is a crucial aspect for the future application of these proteins as anti-yeast compounds.

## Sequence and structure of anti-yeast plant defensins and fungal AFPs

In the 1990s, the first identified plant defensins were isolated from barley and wheat grains, respectively (Colilla et al. 1990; Mendez et al. 1990). These proteins were initially referred to as γ-thionins as their size and cysteine content were similar to the formerly described thionins (Carrasco et al. 1981). However, structure analysis subsequently demonstrated that γ-thionins were related to mammalian and insect defensins and were renamed as plant defensins (Terras et al. 1995). Plants that encode defensins in their genome normally encode more than one. For instance, in the ornamental tobacco *Nicotiana alata,* two plant defensins NaD1 and NaD2 have been identified and extensively studied (Dracatos et al. 2016, 2014; Hayes et al. 2013; Lay et al. 2003). Similarly, in the radish *Raphanus sativus*, two defensins RsAFP1 and RsAFP2 have long been described and well characterized (Aerts et al. 2009, 2007; Tavares et al. 2008; Thevissen et al. 2012; Vriens et al. 2016). The diversity and function of plant defensins have been extensively reviewed elsewhere (Kovaleva et al. 2020; Parisi et al. 2019b).

After the early sequencing of the anti-fungal AFP from *Aspergillus giganteus* (Nakaya et al. 1990), the PAF from *Penicillium chrysogenum* was identified as an abundantly secreted, small, cationic protein (Marx et al. 1995), and has been broadly studied and characterized. Filamentous ascomycetes that encode AFPs in their genomes contain from 1 to 3 phylogenetically distinct *afp* genes, although not necessarily produce the corresponding proteins (Garrigues et al. 2016). In fact, the production of PAFB and PAFC proteins from *P. chrysogenum* has been recently achieved only under certain growing conditions and with relatively low yields (Holzknecht et al. 2020; Huber et al. 2019). The fruit pathogen *P. expansum,* for instance, also encodes three AFPs although natural production only occurs for PeAfpA and PeAfpC in certain growth media, being PeAfpA one of the AFPs with higher production yields and anti-fungal (including anti-yeast) activity of those reported in literature (Gandia et al. 2020; Garrigues et al. 2018). Likewise, *Neosartorya* (*Aspergillus*) *fischeri* only produces two AFPs from the three encoded in its genome, the NFAP and the distantly related and anti-yeast NFAP2 albeit with very low yields and under certain growing conditions (Kovács et al. 2011; Tóth et al. 2016).

**Fig. 1:**
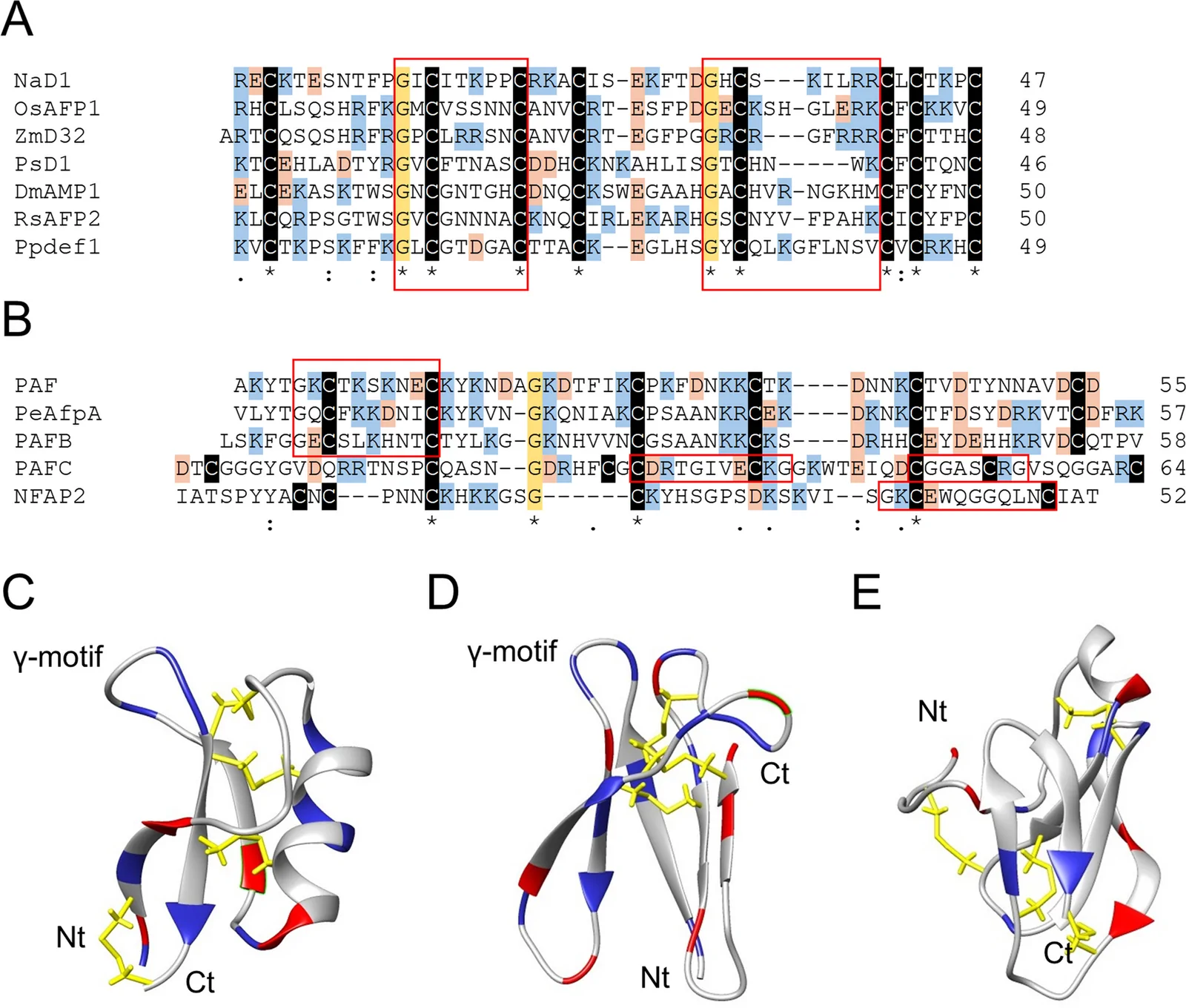
Sequence and structure of plant defensins and fungal AFPs. **A** Alignment of the sequence of the plant defensins NaD1 (UniProt ID: Q8GTM0), OsAFP1 (Q6K209), ZmD32 (B6SJE6), PsD1 (P81929), DmAMP1 (P0C8Y4), RsAFP2 (P30230), and Ppdef1 (van der Weerden et al. 2023). **B** Alignment of the sequence of the fungal AFPs PAF (B6HWK0), PeAfpA (A0A0A2K8K6), PAFB (D0EXD3), PAFC (B6HMF2), and NFAP2 (A0A1D0CRT2). In A and B, alignments were performed using the program Clustal Omega (https://www.ebi.ac.uk/Tools/msa/clustalo/); cysteines are shaded in black, conserved glycines in orange, basic amino acids in blue, and acid amino acids in red; the “asterisk” indicates identical amino acids, “colon” amino acids with strongly similar properties, and “full stop” amino acids with weakly similar properties; and γ-motifs are boxed in red. Ribbon representation of the structure of the plant defensin NaD1 (PDB ID: 1MR4) (**C**) and the anti-fungal proteins PAF (2MHV) (**D**) and PAFC (6TRM) (**E**). Cysteine disulfide bonds are shown in yellow, basic amino acids in blue, and acid amino acids in red. The location of the N-terminus, C-terminus, and the γ-motifs is indicated

Figure 1 shows the sequence and structure of selected plant defensins and fungal AFPs with anti-yeast activity, which are discussed in this review. The plant defensins PsD1 from *Pisum sativum*, DmAMP1 from *Dahlia merckii*, and RsAFP2 from *Raphanus sativus* were originally identified and purified from seeds (Almeida et al. 2000; François et al. 2002). By contrast, NaD1 is purified from the flowers of *Nicotiana alata* (Lay et al. 2003). The other three plant defensins shown in Fig. 1A, which are Ppdef1 from *Picramnia pentandra*, the rice OsAFP1, and the maize ZmD32, were identified in data mining in in silico approaches and produced recombinantly (Kerenga et al. 2019; Ochiai et al. 2018; van der Weerden et al. 2023). In Fig. 1B, the AFPs PAF, PAFB, and PAFC from *P. chrysogenum*, *P. expansum* PeAfpA, and *N. fisheri* NFAP2 are shown.

Plant defensins share an eight cysteine-stabilized CSαβ motif. This motif is formed by a triple-stranded β-sheet linked to an α-helix by three disulfide bonds in the center of the structure and a fourth one formed between the first and the last cysteines, which bind the N- and C-terminal regions and render the protein pseudo cyclic (Almeida et al. 2002; Kovaleva et al. 2020; Parisi et al. 2019b) (Fig. 1C). The structural conservation of these proteins is reflected in the spacing and positions of the eight cysteines found in the amino acid sequence of plant defensins. However, beyond the eight cysteine pattern and the cationic charge of the proteins, no obvious sequence conservation is observed in these proteins as evidenced by the limited sequence identity of the alignment.

Even more sequence and structural variability is found among AFPs, reflecting their less conserved cysteine pattern and higher evolutionary divergence (Fig. 1B, D, E). AFPs contain either six or eight cysteine residues forming three or four disulfide bonds. The more related PAF, PeAfpA, and PAFB have a conserved pattern of six cysteines and fold into a five-stranded β-sheet structure that is maintained by three disulfide bonds (Batta et al. 2009) (Fig. 1D). PAFC shows strong sequence and structural similarity with the previous bubble protein (BP) from *P. brevicompactum* (Czajlik et al. 2021). Both proteins fold into a five-stranded β-sheet preceded by a N-terminal short α-helix with four disulfide bonds, and therefore, their structure diverges from that of the other AFPs (Fig. 1E). NFAP2 is the more distantly related AFP (Sonderegger et al. 2018), and its structure is yet to be solved.

Both plant defensins and fungal AFPs share the presence of the so-called γ-motif, a structural motif with the consensus sequence X_3_GXC[x]_3-9_C that folds into two connected β-sheets identified in proteins with anti-microbial activity (Yount and Yeaman 2004) (Fig. 1). Although plant defensins contain two γ-motifs, only the C-terminal one in its dextromeric isoform has been shown to contain determinants of anti-fungal activity (Sagaram et al. 2011). In the closely related PAF, PAFB, and PeAfpA, the dextromeric γ-motif is located near the N-terminus. While the γ-motif has been shown to modulate the anti-fungal activity of the PAF protein (Sonderegger et al. 2018), in PAFB it was suggested to be a structural determinant for protein stabilization (Huber et al. 2020). In the distantly related PAFC and NFAP2, two or one potential γ-motifs exist in the second half of the amino acid sequence, respectively, but only in PAFC the central levomeric γ-motif has been shown to contain anti-fungal determinants (Czajlik et al. 2021).

## In vitro potency of plant defensins and fungal AFPs against yeast

Some plant and fungal CRPs exert in vitro anti-fungal activity with inhibitory potencies in the micromolar range against *S. cerevisiae* and *C. albicans*, showing differences in anti-fungal activity depending on the proteins and yeast species, as summarized in Table 1. However, it is worth mentioning that the anti-fungal activity is strongly dependent on the experimental conditions tested, i.e., inoculum dose, microbiological medium used, and remarkably, the ionic strength of the medium due to the prominent cationic character of these CRPs. Therefore, it is difficult to compare experiments conducted in different laboratories.

**Table 1:** In vitro potency of plant defensins and AFPs against *S. cerevisiae* and *C. albicans*

CRP	Origin	MIC (µM)		Reference
		*S. cerevisiae*	*C. albicans*	
Defensin				
ApDef1	*Adenanthera pavonina*	7.8	-	Soares et al. (2017)
DmAMP1	*Dahlia merckii*	0.32	5	Bleackley et al. (2019) and Thevissen et al. (2004)
HsAFP1	*Heuchera sanguinea*	3.4	-	Aerts et al. (2011)
NaD1	*Nicotiana alata*	2.5	2.5–5	Bleackley et al. (2014), Hayes et al. (2013), Shahmiri et al. (2023)
NaD2	*Nicotiana alata*	-	5	Shahmiri et al. (2023)
NbD6	*Nicotiana benthamiana*	3 (IC_70_)	-	Parisi et al. (2019a)
OsAFP1	*Oryza sativa*	4–16	4	Ochiai et al. (2018)
Ppdef1	*Picramnia pentandra*	1.7 (IC_50_)	5.7	Parisi et al. (2024) and van der Weerden et al. (2023)
PsD1	*Pisum sativum*	-	20	Gonçalves et al. (2017)
Purple pole bean defensin	*Phaseolus vulgaris*	-	4.8	Lin et al. (2009)
PvD1	*Phaseolus vulgaris*	-	> 9.2 (IC_50_)	Games et al. (2008)
RsAFP2	*Raphanus sativus*	-	2.5	Thevissen et al. (2004)
SbI6	*Glycine max*	5 (IC_70_)	-	Parisi et al. (2019a)
TsD10	*Taraxacum* spp.	20	-	Bleackley et al. (2019)
ZmD32	*Zea mays*	-	2.5	Shahmiri et al. (2023)
AFP				
AnAFP	*Aspergillus niger*	8	8–15	Lee et al. (1999)
NFAP2	*Neosartorya fischeri*	0.04–0.56	0.14–1.12	Tóth et al. (2016) and Tóth et al. (2018)
PAF	*Penicillium chrysogenum*	2	4	Huber et al. (2020) and Sonderegger et al. (2018)
PAFB	*Penicillium chrysogenum*	1	1	Huber et al. (2020) and Huber et al. (2018)
PAFC	*Penicillium chrysogenum*	-	2.5	Holzknecht et al. (2020)
PAF^var^	Rational design	-	1.3	Sonderegger et al. (2018)
PAF^opt^	Rational design	-	1.3	Sonderegger et al. (2018)
PeAfpA	*Penicillium expansum*	0.6–1.2	1.2	Garrigues et al. (2018) and Giner-Llorca et al. (2023a)

*-*: no data available, *MIC*: minimum inhibitory concentration, *IC*_*50*_: concentration that inhibits 50% of growth, *IC*_*70*_: concentration that inhibits 70% of growth

Minimum inhibitory concentration (MIC) values of defensins against *S. cerevisiae* range from 0.32 to 20 µM. The lowest MIC value corresponds to the salt-tolerant DmAMP1, which is able to maintain its in vitro potency even in the presence of 100 mM NaCl (Bleackley et al. 2019). Regarding CRP potencies against *C. albicans*, the range of MICs vary from 2.5 to 20 µM. Of note is the radish defensin RsAFP2, which acts synergistically with caspofungin and amphotericin B in the prevention and eradication of *C. albicans* biofilms (Vriens et al. 2016). Additionally to RsAFP2, RsAFP1 and HsAFP1 were also shown to reduce the biofilm-forming capability of *C. albicans* (Vriens et al. 2016, 2015). Defensins able to inhibit the growth of both yeast species are DmAMP1 (Bleackley et al. 2019; Thevissen et al. 2004), NaD1 (Bleackley et al. 2014; Hayes et al. 2013), OsAFP1 (Ochiai et al. 2018), and Ppdef1 (Parisi et al. 2024; van der Weerden et al. 2023).

In addition, some defensins also display activity towards other relevant pathogenic yeasts. Defensins active against other *Candida* species are ZmD32 and Ppdef1, which inhibit the growth of *Candida auris*, *Candida glabrata*, *Candida krusei*, and *Candida tropicalis* (Kerenga et al. 2019; Parisi et al. 2024); PvD1 with activity towards the two latter and also against *Candida guilliermondii* (Games et al. 2008); HsAFP1 with activity against *C. krusei* (Thevissen et al. 2007); and DmAMP1, which is effective towards *C. glabrata* (Thevissen et al. 2007). Besides, the defensins NaD1 and Ppdef1 exert anti-fungal activity against several *Cryptococcus* species at low micromolar concentrations (Hayes et al. 2013; Parisi et al. 2024).

Regarding AFPs, anti-yeast activity has been described for the three *P. chrysogenum* AFPs (Holzknecht et al. 2020; Huber et al. 2020; Huber et al. 2018; Sonderegger et al. 2018), *N. fischeri* NFAP2 (Tóth et al. 2016, 2018), AnAFP from *Aspergillus niger* (Lee et al. 1999), and PeAfpA from *P. expansum* (Garrigues et al. 2018) (Table 1). In general, AFPs show higher potency towards yeasts than plant defensins. MIC values from AFPs vary between 0.04 and 8 µM and 0.14 and 15 µM towards *S. cerevisiae* and *C. albicans*, respectively. Additionally, PAF^var^ and PAF^opt^, which are rationally designed variants of the *P. chrysogenum* PAF with slight modifications in its γ-core sequence, are significantly more potent against *C. albicans* than the parental protein (Sonderegger et al. 2018) (Table 1).

The highly active protein NFAP2 also inhibits the growth of *C. glabrata, C. guilliermondi, C. krusei, Candida lusitaniae, C. parapsilosis*, and *C. tropicalis* (Tóth et al. 2016, 2018), and recently, it has also been proven effective against *C. auris* with MICs ranging from 5.8 to 92 µM (Kovacs et al. 2021). PAFC has also been described as a potent anti-*Candida* protein, since in addition to inhibiting the growth of *C. albicans,* it exerts anti-fungal activity against *C. glabrata, C. parapsilosis, C. guilliermondii*, and *C. krusei* (Holzknecht et al. 2020). Additionally, the anti-biofilm activity of PAFC has also been reported (Holzknecht et al. 2020). Similarly, PeAfpA also displays activity against other *Candida* species such as *C. glabrata* and *C. parapsilosis* (Garrigues et al. 2018).

## In vivo application of plant defensins and fungal AFPs against yeast infections

Due to a generally poor correlation between the in vitro and in vivo activities of anti-fungals, which may be due to factors such as drug pharmacokinetics, drug delivery to the infection site, and host response to each anti-fungal agent, there is a clear need for further development of relevant in vivo assays. In this sense, animal models — or alternatively three-dimensional (3D) tissue equivalent models — are very good candidates to test new drugs, and to ensure their safety before moving into the clinical phase with human subjects (Holzknecht et al. 2022; Thevissen et al. 2007). Although plant defensins and fungal AFPs are potential candidates for the treatment of yeast infections, many of them either (i) lack information on their in vivo anti-fungal potency or (ii) fail when advancing to in vivo testing. In this regard, several approaches are being applied to overcome these obstacles, for example, the rational design of peptides with amino acid substitutions that can confer greater anti-fungal activity in vivo and less toxicity to the host cells (Torres et al. 2021).

So far, very few plant defensins and AFPs have undergone in vivo testing against pathogenic yeasts, with *Candida*-related diseases accounting for the majority of in vivo applications reported to date (Table 2). Nevertheless, plant defensins are more advanced in this sense and are already entering clinical trials as treatments for fungal-related infections, as it is the case of Ppdef1 as a topical treatment for fungal nail diseases caused by *Candida* spp. among other fungi (Hein et al. 2022). Other example of in vivo application of plant defensins is RsAFP2, which was shown to reduce the fungal burden 5 days after *C. albicans* infection with a prophylactic administration of this peptide in murine models (Tavares et al. 2008). Additionally, PvD1 prolonged the survival rate of *Candidiasis-*infected *Galleria mellonella* larvae without causing any toxic effects on the insect (Skalska et al. 2020). Remarkably, PvD1 showed even higher anti-fungal effect on *C. albicans*-infected larvae than the standard anti-mycotic drug amphotericin B.

**Table 2:** Ex vivo and in vivo applications of plant defensins and AFPs against *Candida* spp

CRP	Target yeast	Dose	Application	Reference
Defensin				
Ppdef1	*Candida* spp.	-	Onychomycosis	Hein et al. (2022) and van der Weerden et al. (2023)
PvD1	*C. buinensis* *C. tropicalis* *C. albicans* *C. parapsilosis*	100 μg/mL, (18.35 μM)	*Galleria mellonella* candidiasis	Skalska et al. (2020)
RsAFP2	*C. albicans*	14 mg/kg	Murine candidiasis	Tavares et al. (2008)
AFP				
NFAP2	*C. albicans*	800 μg/mL/day	Murine vulvovaginitis	Kovács et al. (2019)
		160 μg	3D skin infection model	Holzknecht et al. (2022)
PAF^opt^	*C. albicans*	440.31 μg	3D skin infection model	Holzknecht et al. (2022)
PAFB	*C. albicans*	440.31 μg	3D skin infection model	Holzknecht et al. (2022)
PAFC	*C. albicans*	440.31 μg	3D skin infection model	Holzknecht et al. (2022)

*-*: no data available

Regarding the in vivo efficacy of fungal AFPs against infectious yeasts, *N. fisheri* NFAP2 has been demonstrated to significantly potentiate the inhibitory effect of traditional anti-fungals such as fluconazole, amphotericin B, or caspofungin against the biofilm-forming ability of *C. auris* (Kovacs et al. 2021). Furthermore, the therapeutic potency of NFAP2 as a topical agent has been proven in combination with fluconazole for the treatment of vulvovaginal candidiasis caused by *C. albicans* in a murine model without causing morphological alterations in the vaginal and vulvar tissues (Kovács et al. 2019). Additionally, NFAP2 as well as the *P. chrysogenum* PAF^opt^, PAFB, and PAFC diminished the fungal burden and penetration depth of *C. albicans* in an infected 3D full-thickness skin model, restoring the original epidermal permeability barrier and decreasing the secretion of the pro-inflammatory chemokine IL-8 upon AFP treatment (Holzknecht et al. 2022).

## Mode of action of plant defensins and fungal AFPs with anti-yeast activity

Mechanisms of action of plant defensins and fungal AFPs are more complex than simple membrane permeabilization induced by many small AMPs. They show a multi-target mechanism of action different from those of the traditional anti-fungals, making fungal isolates less likely to overcome their inhibitory action, thus decreasing the likelihood of resistance. This scenario has been confirmed in a study comparing the development of resistance to caspofungin and to the plant defensin NaD1 (McColl et al. 2018).

Mechanisms of action include different targets ranging from interaction with the cell wall (CW) and plasma membrane, which in some cases could lead to direct membrane permeabilization, to exerting their action internally. The first step in the mode of action of anti-fungal peptides is the physical interaction with the outer structures that surround microbial cells. In general, their cationic nature allows an electrostatic attraction towards the negatively charged microbial envelopes, where specific components located in the CW and/or the plasma membrane of target fungi aid in the interaction (Marcos et al. 2008; Muñoz et al. 2013).

Some plant defensins target distinct fungal membrane lipids of yeast and filamentous fungi (Neves de Medeiros et al. 2014; Ramamoorthy et al. 2007; Thevissen et al. 2003, 2004). Glucosylceramide (GlcCer) is the simplest glycosphingolipid, from which a great diversity of glycolipids from the plasma membrane of fungi, plants, and animals are derived. The radish defensin RsAFP2 interacts with GlcCer in the plasma membrane of susceptible *C. albicans* and *Komagataella phaffii* (formerly known as *Pichia pastoris*), and this interaction leads to a subsequent permeabilization and cell growth arrest (Thevissen et al. 2004). Mutant strains of these species lacking GlcCer or *S. cerevisiae* and *C. glabrata* lacking GlcCer in their membranes are resistant to RsAFP2 (Thevissen et al. 2004). In susceptible *C. albicans*, RsAFP2 induces endogenous reactive oxygen species (ROS), but not in the RsAFP2-resistant mutant lacking GlcCer (Aerts et al. 2007). The highly similar AFP1 from *Brassica juncea* does not inhibit mutants of *C. albicans* lacking a specific methyl group in the GlcCer sphingoid base moiety, and this lack of activity is correlated with the absence of ROS production (Oguro et al. 2014). Interestingly, the *P. sativum* defensin Psd1 preferentially binds to vesicles containing GlcCer isolated from fungi as opposed to vesicles formed with GlcCer from plants, thus supporting a specificity for distinct GlcCer (Neves de Medeiros et al. 2014).

Membrane sphingolipids are another class of lipids that are targets for plant defensins*.* Genes determining the sensitivity of *S. cerevisiae* towards DmAMP1 were identified as *IPT1* and *SKN1*, involved in the biosynthesis of the sphingolipid mannosyldiinositol phosphorylceramide (Thevissen et al. 2000, 2005). Sensitivity to DmAMP1 depends on the presence of this sphingolipid in specific ergosterol-containing lipid domains of the plasma membrane (Im et al. 2003). The binding of DmAMP1 to the sphingolipid induces increased K^+^ efflux and Ca^2+^ uptake, as well as membrane potential changes (Thevissen et al. 2000). A search for additional genes that confer sensitivity to DmAMP1 concluded that the defensin may activate the pheromone response pathway after interaction with sphingolipids in the plasma membrane (Parisi et al. 2019a).

Additional support for the relevance of the binding to membrane lipids in the activity of plant defensins to yeast and filamentous fungi arises from in vitro binding studies using protein–lipid overlay assays (Ochiai et al. 2020; Poon et al. 2014; Sagaram et al. 2013). NaD1 was shown to bind several phospholipids including the relevant phosphatidylinositol 4,5-bisphosphate (PIP2), but not other membrane lipids or sphingolipids (Poon et al. 2014). The rice OsAFP1 also binds phosphatidylinositols, although the preferred lipid seems to be phosphatidylinositol 3-phosphate (PI(3)P) (Ochiai et al. 2020). Importantly, the crystal structure of NaD1 bound to PIP2 demonstrated that the bound phospholipid mediates the oligomerization of the defensin in an arrangement of seven dimers that complex 14 molecules of PIP2 (Poon et al. 2014). The crystal structure of the rice OsAFP1 showed a dimeric conformation compatible with that of NaD1 (Ochiai et al. 2020).

Following the binding to *C. albicans* cell surface, NaD1 permeabilizes the membrane and is internalized into the fungal cells, causing killing by a mechanism that, at least in part, depends on oxidative damage through the production of ROS and nitric oxide (NO) (Hayes et al. 2013). In accordance with these results, *S. cerevisiae* [rho^0^] mutants with decreased mitochondrial function and decreased ROS production are more resistant to NaD1 treatment (Hayes et al. 2013). It was shown that the mechanism by which NaD1 is internalized into *C. albicans* cells is the energy-dependent process of endocytosis (Hayes et al. 2018). Other defensin that is internalized as part of its anti-fungal mechanism is PsD1 (Lobo et al. 2007; Neves de Medeiros et al. 2014), for which it was demonstrated that the lack of GluCer blocks internalization in *C. albicans* and reduces, but not abolishes, the anti-yeast activity (Neves de Medeiros et al. 2014).

The oxidative stress produced by ROS is one of the markers of regulated cell death via apoptosis. RsAFP2 induces apoptosis in a metacaspase independent way in *C. albicans* as part of its anti-fungal action (Aerts et al. 2009). OsAFP1 also induces apoptosis in *C. albicans* cells as demonstrated by apoptosis markers (Ochiai et al. 2018). Other plant defensins such as HsAFP1 and PvD1 also kill *C. albicans* by oxidative damage related to induction of ROS and NO production (Aerts et al. 2011; Mello et al. 2011).

Additional valuable information on the mode of action of plant defensins comes from large-scale screenings of collections of mutants. The screening of *C. albicans* mutants for altered RsAFP2 sensitivity showed that the defensin induces CW stress, provokes the accumulation of long-chain ceramides in the plasma membrane, and impairs the yeast to hyphal transition (Thevissen et al. 2012). Regarding NaD1, the screening of a mutant collection of *S. cerevisiae* supported the roles of mitochondria and polyamine transport in the defensin activity (Bleackley et al. 2014; Parisi et al. 2019a). With respect to polyamine transport, the gene *agp2* encoding the cell membrane regulator of polyamine and carnitine transport Agp2p is of particular interest. Deletion of the *agp2* gene confers tolerance to NaD1 via a mechanism that includes diminished defensin internalization (Bleackley et al. 2014). A similar screening for altered sensitivity of *S. cerevisiae* towards HsAFP1 identified genes implicated in different functions including (i) vacuolar acidification and protein sorting/vesicular transport, (ii) gene expression/DNA repair, (iii) mitochondrial function, (iv) cytoskeletal organization and cytokinesis, (v) CW biosynthesis and maintenance, and (vi) stress response signaling (Aerts et al. 2011). An important part of genes involved in HsAFP1 mode of action were found to be implicated in mitochondrial functionality, as described for NaD1 (Bleackley et al. 2014; Parisi et al. 2019a). Moreover, authors demonstrated that HsAFP1-treated *C. albicans* cultures accumulate ROS and exhibit key markers of apoptosis, suggesting the induction of mitochondrion-dependent apoptosis by HsAFP1 in susceptible yeasts. Another screening of the *S. cerevisiae* non-essential gene deletion mutants also highlighted the role of the mitochondria in the mechanism of action of Ppdef1 (Parisi et al. 2024). The defensin rapidly enters *S. cerevisiae* cells, causing a rapid hyperpolarization of the mitochondrial membrane and cellular death. Authors also demonstrated vacuole fusion and ROS production prior to plasma membrane disruption and cell death (Parisi et al. 2024).

Finally, the toxic effect of the two related defensins NbD6 from *Nicotiana benthamiana* and the soybean SBI6 is dependent on a properly functioning vacuolar system (Parisi et al. 2019a). This result was based on the observation that *S. cerevisiae* strains with deletions in vacuolar genes have increased tolerance to NbD6 and SBI6, confirmed by confocal microscopy. Since there were yeast strains only resistant to either NbD6 or SBI6, authors hypothesized the existence of additional determinants and a similar — but not equal — involvement of the vacuole in the mechanism of action of both defensins. Moreover, several strains with mitochondrial defects showed increased resistance to NbD6 in accordance with the induction of ROS after defensin treatment. By contrast, there was a lack of ROS production after treatment with SBI6.

Although the activity of several fungal AFPs against different *Candida* species and *S. cerevisiae* has been described, their anti-yeast mechanism of action is not as characterized as in the case of plant defensins. It is known that the three *P. chrysogenum* proteins, PAF, PAFB, and PAFC, require uptake and cytoplasmic localization before plasma membrane permeabilization occurs, pointing towards the existence of intracellular targets (Holzknecht et al. 2020; Huber et al. 2020; Huber et al. 2018). Studies also corroborate that the mode of action of the three *P. chrysogenum* AFPs is closely linked with ROS production not only in filamentous fungi but also in yeast cells (Holzknecht et al. 2020; Huber et al. 2020; Huber et al. 2018; Sonderegger et al. 2018), suggesting oxidative stress as part of a broad killing mechanism shared with most of the anti-yeast proteins described in this review. It is important to note that all these studies were conducted at protein concentrations well-above the MIC in each protein-microorganism combination.

PeAfpA at sub-inhibitory concentrations first interacts with the outer envelope of *S. cerevisiae* cells and then translocates to the cytoplasm, prior to cell permeabilization and killing (Giner-Llorca et al. 2023b). PeAfpA enters the cell not only by an active energy-dependent (endocytic-like) mechanism but also by passive diffusion. Moreover, microscopy studies indicated that internalization by itself does not provoke permeabilization or cell death, and suggested that PeAfpA does not damage CW or plasma membrane structures when enters the cell. As occurs with some plant defensins described above, PeAfpA binds membrane phospholipids in vitro (Giner-Llorca et al. 2023a). However, comparison studies with different AFPs and chimeric proteins with different degrees of activity suggest that there is not a direct correlation between phospholipid binding and anti-fungal activity.

The main anti-fungal mechanism of the highly effective anti-yeast protein NFAP2 seems to be the disruption of the plasma membrane, based on the fact that this AFP was not able to cause metabolic inactivity and apoptosis induction in susceptible *S. cerevisiae* cells (Tóth et al. 2016). This plasma membrane disruption effect was also observed in *C. albicans* cells (Kovács et al. 2019; Tóth et al. 2018). Scanning electron microscopy images showed that NFAP2 causes alterations in the surface of *C. albicans* cells (Kovács et al. 2019). Authors hypothesized that the presence of a fungus-specific plasma membrane target may be involved in the anti-fungal mechanism of NFAP2, although this target has not been identified yet.

Novel clues about the mode of action of PeAfpA were obtained by combining transcriptional profiling, screening of *S. cerevisiae* mutants with altered PeAfpA sensitivity, and microscopy studies (Giner-Llorca et al. 2023b). This study unveils similarities but also differences in the mode of action of different AFPs and plant defensins. PeAfpA at sub-inhibitory concentration induces global stress, affects distinct signaling routes, and changes the expression of CW-related genes (Giner-Llorca et al. 2023b). All the three mitogen-activated protein kinase (MAPK) signaling routes and the cyclic adenosine monophosphate–protein kinase A (cAMP-PKA) pathway were affected by PeAfpA, but with distinct contributions. Thus, null mutants of the MAPK CW integrity pathway and the cAMP-PKA signaling were among the most tolerant to PeAfpA. On the other hand, mutants in the high-osmolarity glycerol (HOG) and the filamentation-invasion (KSS1) MAPK pathways were among the most sensitive, indicating a role in the yeast defense against the protein. In the case of plant defensins acting against *Candida*, mutants in the HOG pathway were similarly more sensitive to NaD1 and DmAMP1, while other signaling pathways had no effect (Hayes et al. 2013).

In addition, the two yeast mutants most susceptible to PeAfpA were those with the *VPS34* and *SAC1* genes mutated. These genes encode phosphatidylinositol metabolism-related proteins involved in protein sorting and endocytic processes, thus connecting the involvement of specific phospholipids, endocytosis, and protein trafficking in the mode of action of PeAfpA. Additionally, the mutation of the *END3* gene that is required for proper endocytic internalization also resulted in increased tolerance to PeAfpA, further supporting the role of endocytosis in the PeAfpA mode of action. However, and in contrast to NaD1 (Bleackley et al. 2014), deletion of the *AGP2* gene that codes for a regulator of polyamine uptake did not confer tolerance to PeAfpA, confirming that both CRPs show differences in their mode of action.

## Biotechnological production

Commercialization of anti-fungal proteins with anti-yeast activity, both naturally occurring and rationally designed, requires stable, cost-effective production to ensure sufficient amounts of proteins of adequate quality and purity. Development of plant defensins and AFPs for medical or biotechnological purposes requires large amounts of purified peptides. However, usually very low (or even no) yields are obtained from their native producers (Table 3), with this alternative being restricted to natural peptides and not to those obtained, e.g., by rational design (Vriens et al. 2014), thus limiting the application of these proteins as anti-yeast compounds. In this sense, both chemical synthesis and recombinant production can be applied to overcome the problematic of natural production. Although the synthetic production might be affordable in the case of clinical use of these proteins, their size and particular tertiary structure make biotechnological production the best commercially viable alternative (Thevissen et al. 2007). These proteins have been heterologously produced in different hosts such as bacteria, yeasts, filamentous fungi, and plants (Table 3). Protein production by the bacterium *Escherichia coli* offers some advantages due to its easy and cost-effective cultivation. However, this producing system presents some disadvantages for the expression of functional defensins and AFPs: (i) codon bias when expressing eukaryotic genes; (ii) need for protein toxicity neutralization; (iii) incorrect disulfide bridge formation; and (iv) inclusion body formation, which complicates further protein purification steps (Sonderegger et al. 2016; Vriens et al. 2014). In literature, there are very few examples of plant defensins with anti-yeast activity that have been successfully produced in *E. coli* (Table 3)*,* being those restricted to DmAMP1 (Parisi 2017), OsAFP1 (Ochiai et al. 2018), and NaD1 (Bleackley et al. 2016). In the case of fungal AFPs, no anti-yeast AFPs have been produced in bacteria yet, although other AFPs, e.g., *Aspergillus giganteus* AFP (Chen et al. 2023) or *Monascus pilosus* MAFP1 (Tu et al. 2016), have been successfully produced in this biofactory. Therefore, although there are a few examples that would validate this expression system for the potential production of defensins and AFPs with anti-yeast activity, there are alternative expression systems when producing proteins with high cysteine content.

**Table 3:** Production of plant defensins and fungal AFPs with anti-yeast activity

CRP	Natural production	Heterologous organism	Production method	Recombinant production	Reference
Defensin					
DmAMP1	n.d	*E. coli*	pHUE	5 mg/L	Parisi (2017)
		*K. phaffii*	pPIC9/pPINK	Low	Hayes et al. (2013) and Parisi (2017)
		*Arabidopsis*	pFAJ3105	0.62% total soluble protein in leaf-derived crude extracts	François et al. (2002)
HsAFP1	n.d	*K. phaffii*	pPICZαA	40 mg/L	Cools et al. (2017) and Vriens et al. (2015)
NaD1	2.3% total protein extract	*E. coli*	pHUE	n.d	Bleackley et al. (2016)
		*K. phaffii*	pPIC9	3.2 mg/L	Dracatos et al. (2014) and van der Weerden and Anderson (2015)
NbD6	-	*K. phaffii*	pPINK	n.d	Kerenga et al. (2019)
OsAFP1	-	*E. coli*	pGEX-6p-1	n.d	Ochiai et al. (2018) and Ochiai et al. (2020)
PaD2	-	*K. phaffii*	pPINK	n.d	Kerenga et al. (2019)
Ppdef1	n.d	*K. phaffii*	pPIC9	6.3 mg/L	van der Weerden and Anderson (2015)
PsD1	0.5% total seed protein content	*K. phaffii*	pPIC9	13.8–63 mg/L	Almeida et al. (2000), Almeida et al. (2001), Cabral et al. (2003)
RsAFP2	30 mg/kg of seeds	*K. phaffii*	pPIC9/ pPICZαA	100 mg/L	Spelbrink et al. (2004), Terras et al. (1992), Vriens et al. (2016)
		*Arabidopsis*	pFAJ3105	0.15% total soluble protein in leaf-derived crude extracts	François et al. (2002)
ZmD32	-	*K. phaffii*	pPINK	n.d	Kerenga et al. (2019)
AFP					
NFAP2	0.37 mg/L	*P. chrysogenum*	*paf* cassette	15 mg/L	Tóth et al. (2016) and Tóth et al. (2018)
PAF	High amount	*P. chrysogenum*	*paf* cassette	80 mg/L	Batta et al. (2009), Marx et al. (1995), and Sonderegger et al. (2016)
		*P. digitatum*	*paf* cassette	83 mg/L	
		*K. phaffii*	pPic9K	n.d	
PAF^opt^	n/a	*P. chrysogenum*	*paf* cassette	2 mg/L	Sonderegger et al. (2018) and Tóth et al. (2020)
PAF^var^	n/a	*P. chrysogenum*	*paf* cassette	n.d	Sonderegger et al. (2018)
PAFB	0–5 mg/L	*P. chrysogenum*	*paf* cassette	61 mg/L	Huber et al. (2018) and Huber et al. (2019)
PAFC	Low amount	*P. chrysogenum*	*paf* cassette	105 mg/L	Holzknecht et al. (2020)
PeAfpA	125 mg/L	*P. chrysogenum*	*paf* cassette	Low amount	Garrigues et al. (2018)
			*afpA* cassette	5 mg/L	Gandía et al. (2022)

*n.d.*: not determined. *n/a*: not applicable. *–*: no information available

Yeasts have been largely used for production of recombinant proteins — including defensins and AFPs — due to their eukaryotic nature, being able to implement many post-translation modifications, e.g., disulfide bonds, glycosylation, and signal sequence processing, which are crucial for protein functionality (Vriens et al. 2014). Among yeasts, *K. phaffii* is of particular interest for large-scale productions of recombinant proteins, as it can easily grow to ultra-high cell densities in biofermenters, which leads to increased protein yields (Vriens et al. 2014). There are several examples of anti-yeast plant defensins that have been heterologously produced in *K. phaffii*, although for many of them, no recombinant production yields have been reported (Table 3). As examples, yields ranging from 3.2 in the case of NaD1 to 100 mg/L for RsAFP2 were obtained using this yeast as biofactory (Cabral et al. 2003; Cools et al. 2017; Vriens et al. 2016). Anti-yeast AFPs have been mostly biotechnologically produced using filamentous fungi, particularly *P. chrysogenum* and *P. digitatum* as cell factories. In these biofactories, a *P. chrysogenum*-based expression system based on the strong *paf* promoter, signal peptide, and terminator sequences (*paf* cassette) (Sonderegger et al. 2016) was developed for the optimal production of PAF, PAF^opt^, PAF^var^, PAFB, PAFC, and NFAP2, while a *P. expansum*-based expression system based on the strong *afpA* promoter, signal peptide, and terminator sequences (*afpA* cassette) (Gandía et al. 2022) was developed for the recombinant production of PeAfpA in the non-mycotoxigenic fungus *P. chrysogenum*. Overall recombinant protein yields ranged from 2 to 105 mg/L (Table 3), with both systems representing a great tool for cost-effective production of AFPs in generally high yields.

Finally, plants are one of the least developed biofactories for the production of anti-yeast defensins and AFPs. In fact, the heterologous expression of defensins (and AFPs to a lesser extent) in plants is not mainly intended for the biotechnological production of these proteins in high yields, but rather for the acquisition of resistance/tolerance against pathogenic fungi (Coca et al. 2004; Gaspar et al. 2014; Jha and Chattoo 2010; Zhu et al. 2007). Nevertheless, some anti-yeast defensins and AFPs have been biotechnologically produced in plants, such as the defensins DmAMP1 and RsAFP2, which were produced as chimeric polyproteins that were finally cleaved to yield the single protein monomers in *Arabidopsis* (François et al. 2002), or the AFP PeAfpA produced in *Nicotiana benthamiana* through a disarmed viral vector (Manzanares and Marcos, unpublished data). However, the time-consuming processes to obtain transgenic plant lines, along with the complex purification procedures of these anti-fungal proteins from plant tissues, make plants a less desirable biofactory for their bulk production.

## Conclusions and future prospects

Small CRPs from plants and filamentous fungi represent an untapped natural reservoir of novel anti-microbials. Data reported here demonstrate the high potential of plant defensins and fungal AFPs as promising alternatives to currently applied anti-yeast drugs. Despite the in vitro potency of defensins and AFPs, in vivo evidence of efficacy is still lacking in many of the proteins summarized in this review, limiting the number of these proteins entering clinical trials as treatments for fungal-related infections. Although current studies already demonstrate no cytotoxicity of these proteins to human cells, future studies further investigating toxicology, pharmacodynamics, bioavailability, and efficacy of defensins and AFPs would be beneficial for their future application in clinics.

This review additionally provides relevant examples of the mechanisms of action of plant defensins and AFPs. Although the anti-yeast proteins described here seem to share a broad killing mechanism, this review unveils similarities but also differences in the mode of action of different AFPs and plant defensins. Their multi-faceted mode of action makes these proteins viable candidates to counteract the development of fungal resistance, although the identification of cellular targets is still a challenge for most of these proteins. However, the novel mechanisms reported here and others to be described could pave the way to new classes of anti-fungals with modes of action different to existing ones, an important goal ahead.

Safe and cost-effective biofactories remain crucial for application of defensins and AFPs. This review summarizes different biotechnological platforms for CRP production, although yields are still far from those needed for their use in clinical applications. Development of sustainable biofactories, as well as the development of production scaling processes at an industrial level, is still a challenge to be addressed.

In conclusion, progress has been made in the field of anti-yeast defensins and AFPs. It is expected that in the near future the scientific knowledge will facilitate the use of defensins and AFPs as a new arsenal to improve human health and fight anti-fungal resistance.
